# Identification of *Nocardia* species using matrix-assisted laser desorption/ionization–time-of-flight mass spectrometry

**DOI:** 10.1186/s12014-015-9078-5

**Published:** 2015-03-07

**Authors:** Shunsuke Segawa, Motoi Nishimura, Kazuyuki Sogawa, Sachio Tsuchida, Shota Murata, Masaharu Watanabe, Kazuyuki Matsushita, Katsuhiko Kamei, Fumio Nomura

**Affiliations:** Department of Molecular Diagnosis, Graduate School of Medicine, Chiba University, 1-8-1 Inohana, Chuo Ward, Chiba City, Chiba Prefecture Japan; Division of Laboratory Medicine and Clinical Genetics, Chiba University Hospital, 1-8-1 Inohana, Chuo Ward, Chiba City, Chiba Prefecture Japan; Clinical Proteomics Research Center, Chiba University Hospital, 1-8-1 Inohana, Chuo Ward, Chiba City, Chiba Prefecture Japan; Department of Food Biochemistry, School of Life and Environmental Science, Azabu University, 1-17-71 Fuchinobe, Chuo Ward, Sagamihara City, Kanagawa Prefecture Japan; Medical Mycology Research Center, Chiba University, 1-8-1 Inohana, Chuo Ward, Chiba City, Chiba Prefecture Japan

**Keywords:** *Nocardia*, MALDI-TOF MS, MALDI Biotyper, In-house datebase

## Abstract

**Background:**

The MALDI (matrix-assisted laser desorption/ionization) Biotyper system for bacterial identification has already been utilized in clinical microbiology laboratories as a successful clinical application of protoemics. However, in cases of *Nocardia*, mass spectra suitable for MALDI Biotyper identification are often not obtained if such specimens are processed like general bacteria. This problem is related to the insufficiencies in bacterial spectrum databases that preclude accurate specimen identification. Here, we developed a bacterial processing method to improve mass spectra from specimens of the genus *Nocardia*. In addition, with the new processing method, we constructed a novel in-house bacterial database that combines a commercial database and mass spectra of *Nocardia* strains from the Department of Clinical Laboratory at Chiba University Hospital (DCLC) and the Medical Mycology Research Center at Chiba University (MMRC).

**Results:**

The newly developed method (*Nocardia* Extraction Method at DCLC [NECLC]) based on ethanol-formic acid extraction (EFAE) improved mass spectra obtained from *Nocardia* specimens. The *Nocardia* in-house database at Chiba University Hospital (NDCUH) was then successfully validated. In brief, prior to introduction of the NECLC and NDCUH, 10 of 64 (15.6%) clinical isolates were identified at the species level and 16 isolates (25.0%) could only be identified at the genus level. In contrast, after the introduction, 58 isolates (90.6%) were identified at the species level and 6 isolates (9.4%) were identified at the genus level.

**Conclusions:**

The results of this study suggest that MALDI-TOF (time-of-flight) Biotyper system can identify *Nocardia* accurately in a short time in combination with a simple processing method and an in-house database.

**Electronic supplementary material:**

The online version of this article (doi:10.1186/s12014-015-9078-5) contains supplementary material, which is available to authorized users.

## Background

*Actinomycetes*, which are pathogenic and infectious to humans, include both aerobic and anaerobic bacteria; representative of the former are bacteria of the genus *Nocardia*, and representative of the latter include bacteria of the genus *Actinomyces* [1]. Nocardiosis is an infection involving bacteria of the genus *Nocardia*. These bacteria are often isolated from immunocompromised patients taking immunosuppressants or steroids for the purpose of treating collagen disorders or organ transplantation, suggesting that nocardiosis is primarily an opportunistic infection; however, on rare occasions it is seen in healthy individuals [2,3]. Pneumonia can occur from inhalation of *Nocardia*, and entry through a cut in the skin can cause a skin infection. Hematogenous dissemination to organs throughout the body can also occur; for example, cerebral abscesses due to nocardiosis have been reported. Among bacteria of the *Nocardia* genus, *N. farcinica* and *N. nova* are the most frequently isolated, followed by *N. brasiliensis* and *N. cyriacigeorgica*.

Because antimicrobial susceptibility and pathogenicity differ depending on the species of *Nocardia* [4], from a clinical perspective, the results of bacterial identification should be reported rapidly, similar to the minimum inhibitory concentration. In addition, identification at not just the genus level but the species level as well is needed. To distinguish between the genera of actinomycetes, analyses of the taxonomic and physicochemical properties of the organism are necessary, including analyses of mycolic acid and menaquinone and the sugars and amino acids composing the cell wall. These physicochemical properties can be used to identify species of the genus *Nocardia* [5]. However, identification based on physicochemical properties is time consuming, and the identification procedures are complex. On the other hand, as with other bacteria, classification of *Nocardia* based on the 16S rRNA sequence has been introduced [6] and has simplified species identification, but employing this method in the laboratory remains difficult in routine identification testing.

Recently, the MALDI Biotyper (Bruker Daltonics GmbH, Leipzig, Germany) was developed as a new system for bacterial identification [7-9]. In this system, matrix-assisted laser desorption/ionization–time-of-flight mass spectrometry (MALDI-TOF MS) is used in combination with database software. By collecting mass spectra of the bacteria and comparing these to mass spectra of various bacteria compiled in a database and scoring the match, rapid identification of bacteria has become possible [10-12]. In our clinical laboratory, we used MALDI-TOF MS to identify general bacteria isolated from clinical samples and have reported a high identification rate (91.7%) at the species level [7]. More recently, we reported on direct application of MALDI-TOF mass spectrometry to cerebrospinal fluid for rapid pathogen identification in a patient with bacterial meningitis [13]. However, in the identification of acid-fast bacteria and actinomycetes such as those of the genus *Nocardia*, spectra adequate for identification cannot be obtained using only processes for general bacteria due to the presence of aliphatic acids such as mycolic acid in the cell wall, and sometimes, mass spectra of sufficient intensity for scoring *Nocardia* isolates cannot be obtained at all [14]. In addition, data on only 37 isolates from 32 species of *Nocardia* have been compiled in the database (Bruker Biotyper ver. 3.3.1.0 database), which is insufficient from a practical perspective, and currently, species-level identification is often difficult.

To resolve these problems and improve the precision of *Nocardia* identification, we developed a new method for bacterial extraction. We also created and evaluated an in-house database using *Nocardia* strains stored at the Department of Clinical Laboratory at Chiba University Hospital (DCLC) and the Medical Mycology Research Center in Chiba University (MMRC) and report our findings here.

## Results

### Comparison of the NECLC approach and conventional extraction methods

**Figure 1:**
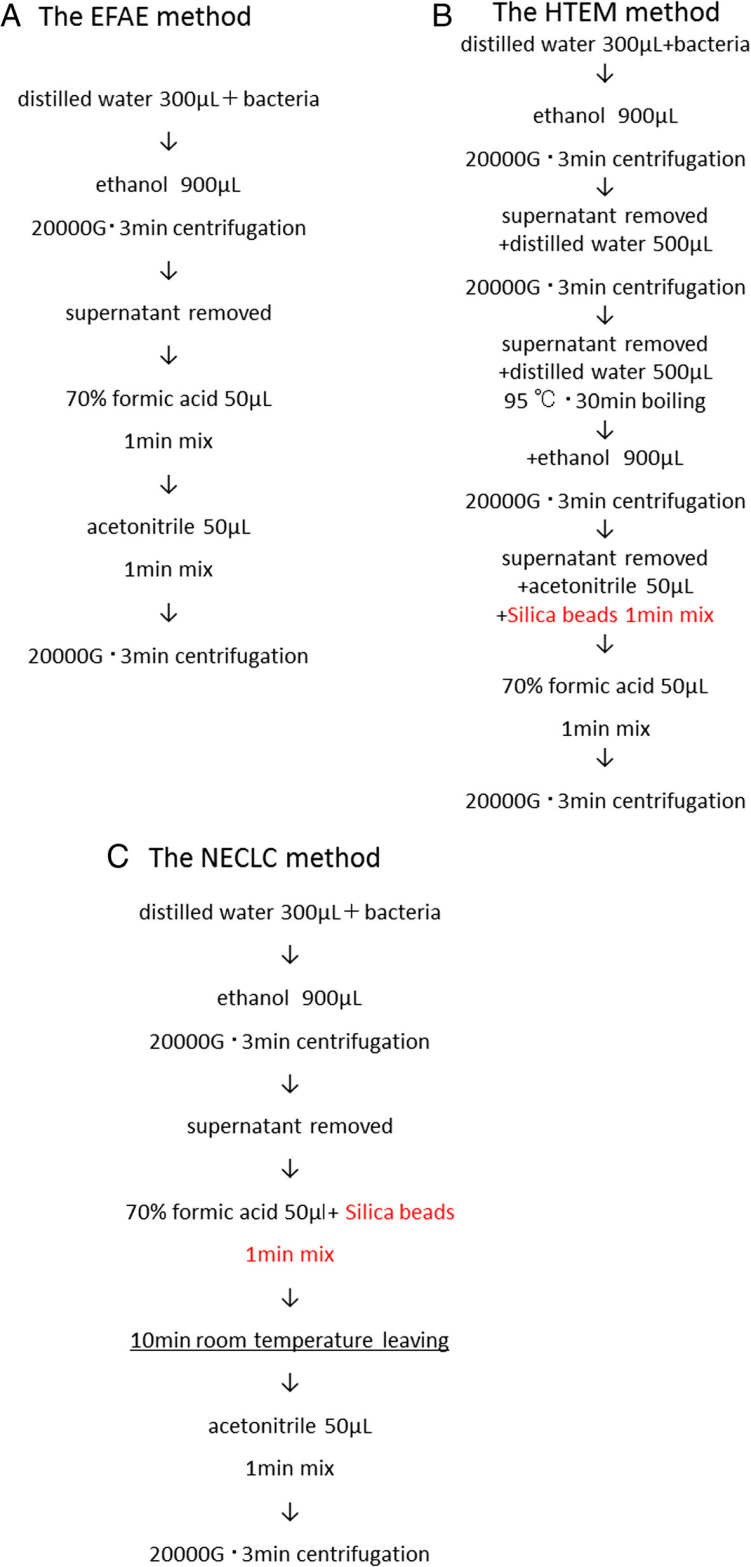
**Comparison of conventional bacterial extraction methods and the newly developed** ***Nocardia*** **extraction method.** Procedures in conventional bacterial sample extraction methods (ethanol-formic acid extraction [EFAE] and high-temperature extraction [HTEM]) are shown in **(A)** and **(B)**, respectively. We propose a new *Nocardia* extraction method (*Nocardia* extraction method in Department of Clinical Laboratory at Chiba University hospital; NECLC). This method is based on EFAE, incorporating an element of the HTEM (indicated in red in the figure) method, with the further addition of a formic acid extraction time of 10 minutes (underlined) **(C)**.

**Figure 2:**
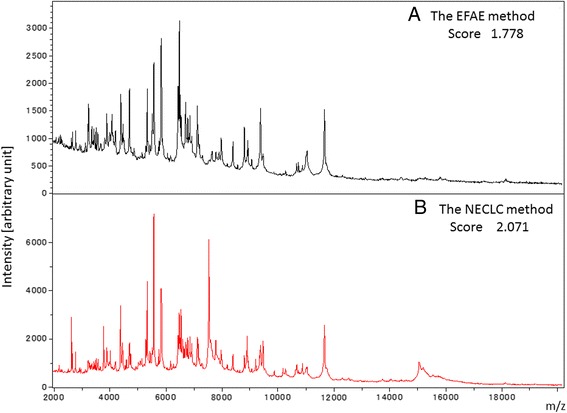
**Representative improvement in spectra of a** ***N. otitidiscaviarum*** **isolate by employing the NELCL method.** Representative spectra and identification scores from analysis of a *N. otitidiscaviarum* isolate using the EFAE method **(A)** and NECLC method **(B)** are shown. Improved spectra and identification scores were obtained using the NECLC approach.

We proposed a new method for *Nocardia* extraction (NECLC). This method is based on the ethanol-formic acid extraction method (EFAE) (Figure 1A), with the addition of silica beads as a component of the high-temperature extraction method (HTEM) (Figure 1B) and use of a 10-minute formic acid extraction time (Figure 1C). Table 1 shows changes in the identification scores of 10 *Nocardia* isolates determined using the NECLC and EFAE methods. Improved identification scores were seen with the NECLC approach, and it became possible to collect mass spectra for bacterial species for which it had previously not been possible. Representative improvement in the spectra is shown in Figure 2. However, species-level identification was still not optimal.

**Table 1:** **Comparison of identification scores for specimens extracted using the EFAE and newly developed NECLC approach**

	**EFAE**	**NECLC**	***P *** **value**
***Nocardia*** **isolate**	**Mean ± SD**	**Mean ± SD**	
*N. nova*-2010/742	1.94 ± 0.06	2.11 ± 0.03	0.00008909^***^
*N. nova*-2011/258	1.89 ± 0.04	2.12 ± 0.04	0.00000340^***^
*N. otitidiscaviarum*-2010/435	1.94 ± 0.09	2.10 ± 0.06	0.00557253^**^
*N. otitidiscaviarum*-2012/744	1.68 ± 0.10	2.01 ± 0.10	0.00001193^***^
*N. farcinica*-2011/1341	1.27 ± 0.13	1.39 ± 0.08	0.02787181^*^
*N. farcinica*-2012/241	1.24 ± 0.08	1.37 ± 0.10	0.02223143^*^
*N. elegans*-2011/259	-	1.28 ± 0.15	#
*N. elegans*-2010/1158	1.22 ± 0.05	1.46 ± 0.10	0.00000972^***^
*N. cyriacigeorgica*-2011/531	-	1.33 ± 0.09	#
*N. cyriacigeorgica*-2012/1454	1.25 ± 0.10	1.40 ± 0.01	0.00209917^**^

Three colonies obtained from each of the various *Nocardia* isolates were analyzed. Each colony was subjected to 3 independent extraction/MS runs, and the identification scores obtained were compared statistically.

*: indicates isolate showing significant improvement (*P* < 0.05). **: *P* < 0.01, ***: *P* < 0.0001, #: indicates sufficient mass spectra for scoring could not be obtained using the EFAE method.

### Validation of the NDCUH

The *Nocardia* in-house database at Chiba University Hospital (NDCUH) was constructed using the new extraction method (NECLC) to process 192 *Nocardia* isolates stored at the MMRC and generate mass spectra as described in the “Methods” section. Results of the cluster analysis were used for the construction of a spectra-based dendrogram (Additional file 1: Figure S5). The *Nocardia* strains were not divided into 2 clusters comprised of the manufacturer’s database strains and the 192 MMRC isolates; they appeared to be distributed without extreme bias.

Next, we evaluated the NDCUH database. In brief, mass spectra were generated for 64 *Nocardia* isolates (Table 2, Additional file 2: Table S1), independent of the isolates used to construct the database, and changes in the mass spectra and identification scores before and after introduction of the in-house database were compared. The results of a comparison of *N. nova* and *N. elegans* isolates are shown in Additional file 3: Figure S6, and the results for 64 *Nocardia* isolates are summarized in Table 3. In brief, prior to introduction of the in-house database, 10 isolates (15.6%) were identified at the species level and 16 isolates (25%) could only be identified at the genus level. In contrast, identification scores improved after introduction of the in-house database, as 58 isolates (90.6%) were identified at the species level and 6 isolates (9.4%) were identified to the genus level only.

**Table 2:** **A summary of the 256** ***Nocardia*** **isolates used in the study**

	**From the MMRC**		**From the DCLC**
	**Used in constructing the in-house database**	**Used in validation**	
No. of isolates	*192	**43	**21

*192 isolates from the MMRC were subjected to MALDI-TOF MS analysis to construct the in-house database, whereas 64 (**43 plus **21) isolates were used for validation of the in-house database. A full detail of identity and characteristics of the 256 *Nocardia* isolates are shown in Additional file 2: Table S1. 192 isolates were from the MMRC and used in constructing the in-house database. 43 isolates were from the MMRC and used in validation. 21 isolates were from the DCLC and used in validation.

MMRC = Medical Mycology Research Center, Chiba University.

DCLC = Department of Clinical Laboratory, Chiba University Hospital.

**Table 3:** **Validation of the NDCUH database**

	**No. of isolates**	**Before the NDCUH introduction**			**After the NDCUH introduction**		
		**Species level**	**Genus level**	***No match***	**Species level**	**Genus level**	**No match**
*N. abscessus*	2			*2*	**1**	1	
*N. aobensis*	2		1	*1*	**2**		
*N. arthritidis*	2			*2*	**1**	1	
*N.a asiatica*	3	**2**	1		**3**		
*N. asteroides*	2			*2*	**2**		
*N. beijingensis*	2			*2*	**2**		
*N. brasiliensis*	2		2		**2**		
*N. concava*	2	**1**		*1*	**1**	1	
*N. cyriacigeorgica*	4			*4*	**4**		
*N. elegans*	4		3	*1*	**4**		
*N. exalbida*	2			*2*	**2**		
*N. farcinica*	7			*7*	**7**		
*N. niigatensis*	2		2		**2**		
*N. nova*	8	**5**	3		**7**	1	
*N. otitidiscaviarum*	3		2	*1*	**3**		
*N. paucivorans*	1	**1**			**1**		
*N. pseudobrasiliensis*	2			*2*	**1**	1	
*N. puris*	3			*3*	**3**		
*N. transvalensis*	2	**1**	1		**1**	1	
*N. veterana*	2		1	*1*	**2**		
*N. vinacea*	2			*2*	**2**		
*N. wallacei*	5			*5*	**5**		
Total	64	**10 (15.6%)**	16 (25.0%)	*38 (59.4%)*	**58 (90.6%)**	6 (9.4%)	*0 (0.0%)*

A total of 64 *Nocardia* isolates, independent of the isolates used to construct the NDCUH, were subjected to identification based on the NECLC method; identification results before and after introduction of the NDCUH database were compared. Before the introduction, the original Bruker Biotyper ver. 3.3.1.0 database was used. In the table, the species level count indicates isolates that could be identified at both the species and genus levels; the genus level count includes not only isolates that could be identified to the genus level but those that could not be identified to the species level.

## Discussion

In the present study, we developed a bacterial processing method (NECLC) and constructed a novel in-house bacterial database (NDCUH) that combines a commercial database and mass spectra of *Nocardia* strains to improve identification of *Nocardia* by MALDI-TOF MS. The NECLC approach improved *Nocardia* identification scores (Table 1), and use of the NDCUH database in conjunction with the NECLC method was then successfully validated (Table 3).

Rapid identification of *Nocardia*, which had been difficult in the past, is now possible thanks to the results of a variety of studies [15-17]. Wauters et al. performed rapid identification using 9 tests, including those involving biochemical properties and enzyme activity, and achieved identification rates of ≥95% for 6 bacterial species commonly isolated from clinical samples [18]. In addition, in a study by Glupczynski et al. *N. farcinica*, *N. nova*, *N. cyriacigeorgica*, *N. abscessus*, and *N. brasiliensis* were identified based on the results of antimicrobial susceptibility testing of 12 species [19]. It is also now possible to identify *Nocardia* by molecular means, and in a study by Conville et al., identification was performed by 16S rRNA genetic analysis [6].

At the MMRC, we typically identify *Nocardia* by determining physicochemical properties and sensitivity to antimicrobials [5]. Genetic analysis of 16S rRNA sequences is also employed at the MMRC. At the clinical laboratory of this hospital, the Mycology Center identifies *Nocardia* isolated from clinical samples, but due to the particulars of identification tests, it takes approximately 1 month for the official identification results to be delivered, which is not compatible with rapid identification.

Bacterial identification using MALDI-TOF MS was developed to replace conventional identification tests. For the genus *Nocardia*, a study by Verroken et al. [14] proposed a processing method that involved high-temperature extraction and their in-house database constructed using 91 isolates from 11 *Nocardia* species. Results of verification of this method using 43 *Nocardia* isolates from 9 species showed that before introduction of their in-house database, 10 isolates (23%) were identified to the species level and 9 isolates to the genus level (21%), whereas after introduction, 34 isolates (79%) were identified to the species level and 4 isolates (9%) could be identified only to the genus level due to improvement in identification scores.

In the study of Verroken et al., a 1-hour pretreatment process that includes 30 minutes of heat treatment was required [14]. The new NECLC approach we propose in this study to replace the EFAE and HTEM methods simplifies the *Nocardia* extraction process and eliminates the requirement for high temperature. As a result, *Nocardia* identification can now be achieved in 30 minutes. We also attempted to enhance the database by incorporating the mass spectra of a variety of bacterial species stored at the Mycology Center. Identification scores improved after introduction of the in-house database, as 58 isolates (90.6%) were identified at the species level and 6 isolates (9.4%) were identified to the genus level only. It is reported that silica beads are effective tools to extract bacterial proteins from acid-fast bacteria [20]. Also, the NECLC method employing silica beads was effective to process *Nocardia* (Additional file 4: Figure S1). Using the proposed NECLC approach and the 192 *Nocardia* isolates from the MMRC, we were able to construct a enriched in-house database (NDCUH), which has contributed to improving identification scores of *Nocardia* species (Table 1, Additional file 3: Figure S6). There were several isolates, however, that could be identified only at the genus level and not the species level. Refinements in the pretreatment procedures and database will be necessary to further improve identification of *Nocardia*.

## Conclusions

Our findings suggest that by using the MALDI-TOF Biotyper MS system along with our new method for bacterial extraction (NECLC) in combination with the in-house database we constructed, the majority of *Nocardia* clinical isolates can now be simply and accurately identified in a short period of time. As a result, the identification rate reached 90.6% at the species level and 9.4% at the genus level.

## Methods

### Culture of Nocardia and strains used

Bacteria were cultured on 5% sheep blood plate agar (Nippon Becton Dickinson Co., Tokyo, Japan) for 3 days at 37°C. The cultures were visually checked for purity, and typical colonies were used to prepare samples for MALDI-TOF MS analysis. In developing the *Nocardia* extraction method described in section 3 below, a total of 10 isolates were used: 2 isolates each of *N. nova*, *N. farcinica*, *N. otitidiscaviarum*, *N. elegans*, and *N. cyriacigeorgica*, all of which had been isolated from clinical samples and stored at the DCLC. To construct the in-house database described in section 4 below, 192 isolates from 73 species of *Nocardia* stored at the MMRC were used (Table 2, Additional file 2: Table S1). For validation of the in-house database as described in section 5 below, a total of 64 *Nocardia* isolates were used: 21 isolates stored at the DCLC and 43 isolates stored at the MMRC that were not used in construction of the in-house database (Table 2, Additional file 2: Table S1). All of the isolates from the DCLC and almost all of the isolates from the MMRC were isolated from clinical samples. At the DCLC and MMRC, we typically identify *Nocardia* by determining physicochemical properties, such as: the ability to degrade organic substances such as adenine, hypoxanthine, and tyrosine; the ability to produce acid from sugars such as glucose, maltose, and mannose; the ability to utilize citric acid, adipic acid, and gluconic acid; and sensitivity to antimicrobials such as imipenem, tobramycin, and ciprofloxacin [5]. Genetic analysis of 16S rRNA sequences is also employed to identify all isolates mentioned above [21-23].

### MALDI biotyper system

A Microflex LT apparatus was used, with the measurement range set at 2 to 20 kDa. FlexControl (ver. 3.3), FlexAnalysis (ver. 3.3), and BioTyper (ver. 3.1) analytical software were used, and version 3.3.1.0 of the database was used for identification. α-CHCA (α-cyano-4-hydroxycinnamic acid) was used as the matrix, and a target plate was used (the apparatus, software, reagents, and database were all products of Bruker Daltonics). The process from MALDI-TOF MS measurement to identification was described in a previous report [7]. In brief, the process was performed automatically without user intervention using the manufacturer’s default settings. The software generated a list of peaks, numbering up to 100. The threshold for peak acceptance was a signal to noise ratio of 10. After alignment, peaks with a mass to charge ratio difference of less than 250 ppm were considered to be identical. The peak list was used for matching against the reference library by direct use of the integrated pattern matching algorithm of the software. Results for the pattern-matching process were expressed as scores ranging from 0 to 3, as suggested by the manufacturer. Scores of <1.7 were not considered to provide reliable identification. A score of ≥1.7 indicated identification of at the genus level, and a score of ≥2.0 indicated identification of at the species level [7].

### Development of the Nocardia extraction method at DCLC (NECLC)

The processes used in the EFAE for general bacteria and the HTEM used for acid-fast bacteria are shown in Figures 1A and 1B. To enable bacterial identification using MALDI-TOF MS, extraction methods have been developed for two decades [20,24,25]. The EFAE method [25] is a simple universal method recommended by the manufacturer for the extraction of general bacteria. The HTEM method [20] is also recommended by the manufacturer for the processing of acid-fast bacteria. To assess the effects of silica beads (Zirconia/Silica Beads 0.5 mm dia; Bio Spec Products inc, USA) used in the attempt to improve the effectiveness of bacterial cell disruption, beads were added during sample extraction in the EFAE method. A 1-μL aliquot of sample was placed on the target plate, dried, overlaid with 1-μL of α-CHCA matrix and dried again, after which the target plate was placed in the MALDI-TOF MS, analyzed, and the changes in identification scores were evaluated. As shown in Additional file 4: Figure S1 and Additional file 2: Table S2, better scores were obtained with the addition of silica beads.

Next, to determine whether to use beads for the EFAE or HTEM method, sample extraction was performed using both methods following the procedures described above, and changes in the identification scores were evaluated. As shown in Additional file 5: Figures S2, Additional file 6: Figures S3 and Additional file 2: Table S3 better scores were obtained when using beads with the EFAE method.

To investigate the effect of varying the duration of formic acid extraction, we extracted samples for 1, 5, and 10 minutes using the same procedures described above and evaluated changes in the identification scores. As shown in Additional file 7: Figure S4 and Additional file 2: Table S4, the best scores were obtained at a formic acid extraction time of 10 minutes.

Based upon all of the above results, we proposed a new method for the extraction of *Nocardia* (the *Nocardia* extraction method in the Department of Clinical Laboratory at Chiba University Hospital; NECLC) (Figure 1C).

### Constructing the in-house database

Samples were extracted from the MMRC’s 192 *Nocardia* isolates using the proposed NECLC approach. The samples were plated (1 μL per site) on 8 sites on the target plate, and after drying, 1 μL of α-CHCA matrix was overlaid onto each sample and then dried. The target plate was placed in the MALDI-TOF MS and each site was analyzed 3 times; thus, with 8 sites, this enabled collection of 24 mass spectra per sample. The resulting data were averaged with FlexControl, and a single mass spectrum representing each sample was generated. The NDCUH was constructed by compiling mass spectra produced by employing this method on 192 isolates and adding these to the manufacturer’s database used for identification as described in section 2 above. Cluster analysis was performed to investigate differences in spectra between strains in the version 3.3.1.0 database and the MMRC’s 192 isolates. The BioTyper (ver. 3.1) software was used to conduct the cluster analysis.

### Validation of the in-house database

Samples were extracted from 64 *Nocardia* isolates (43 isolates from the MMRC and 21 isolates from the DCLC) using the proposed NECLC approach, and mass spectra were then collected. Pattern matching was performed based on each mass spectrum in reference to the NDCUH database, and identification scores before and after introduction of the in-house database were evaluated for improvement.

### Statistical methods

Identification scores are presented in Table 1 as the mean ± SD (standard deviation). The results were analyzed using the paired *t* test for comparisons to determine the significance of differences using the 4-Step Excel Statistics software application (OMS Publishing Inc., Tokorozawa, Japan; http://www.oms-publ.co.jp/index.html). Significance levels were set at a *P* value < 0.05.

## Additional files

Additional file 1: Figure S5. A spectra-based dendrogram of the manufacturer’s database strains and the 192 MMRC *Nocardia* isolates. The BioTyper (ver. 3.1) software was used to perform a cluster analysis and draw the dendrogram. The manufacturer’s database strains are displayed in red, and the MMRC isolates are displayed in black. Additional file 2: Table S1. A full detail of identity and characteristics of the 256 *Nocardia* isolates used in the study. **Table S2.** Improvement in the identification score by incorporating silica beads. **Table S3.** Comparison of identification scores from specimens extracted using the ethanol-formic acid extraction method (EFAE) and high-temperature extraction (HTEM) method. Additional file 3: Figure S6. Representative improvement in mass spectra matching and identification scores using the newly developed extraction method (NECLC) and the in-house database (NDCUH) to identify the same isolates (*N. nova*, *N. elegans*). With the introduction of the NECLC method and NDCUH database, the identification score for *N. nova* increased **(A)**, and *N. elegans*, a strain that was previously unidentifiable, could now be identified to the species level **(B)**. This figure shows matching between the spectrum collected and a reference spectrum stored in the database. Blue indicates the spectrum stored in the database used for pattern matching; in the upper half of the spectrum, green indicates matched peaks, red mismatched peaks, and yellow intermediate peaks. Additional file 4: Figure S1. Comparison of representative mass spectra obtained from extraction with and without silica beads (*N. otitidiscaviarum* isolate). Identification scores determined from each spectrum are indicated. Additional file 5: Figure S2. Comparison of representative mass spectra and identification scores for the same isolate extracted using the HTEM and EFAE methods (*N. farcinica* isolate). Specimens extracted using either the ethanol-formic acid extraction method (EFAE) or the high-temperature extraction method (HTEM) are compared. Additional file 6: Figure S3. Comparison of representative mass spectra and identification scores for the same isolate extracted using the HTEM and EFAE methods (*N. cyriacigeorgica* isolate). Specimens extracted using either the ethanol-formic acid extraction method (EFAE) or the high-temperature extraction method (HTEM) are compared. A baseline rise due to noise was observed between 2,000 to 6000 m/z when the isolate was extracted using the HTEM method, and circled by red line **(A)**. On the other hand, such rise was not observed when using the EFAE method **(B)**. Each mass spectra matching between the spectrum collected and a reference spectrum stored in the database is indicated at the right top corner of each raw spectrum. Blue indicates the spectrum stored in the database used for pattern matching; in the upper half of the spectrum, green indicates matched peaks, red mismatched peaks, and yellow intermediate peaks. Like the baseline noise, mismatched peaks were observed between 2,000 to 6000 m/z when the isolate was extracted using the HTEM method, which may result in a decrease of identification score. Additional file 7: Figure S4. Comparison of representative mass spectra and identification scores for different extraction durations using the EFAE method. Extraction durations of 1, 5, and 10 minutes in the EFAE (ethanol-formic acid extraction) method were tested with the same *N. nova* isolate.

## Abbreviations

MALDI: Matrix-assisted laser desorption/ionization
TOF: Time-of-flight mass
MS: Mass spectrometry
DCLC: Department of Clinical Laboratory at Chiba University Hospital
MMRC: Medical Mycology Research Center at Chiba University
NECLC: *Nocardia* extraction method at DCLC
EFAE: Ethanol-formic acid extraction
HTEM: High-temperature extraction method
NDCUH: *Nocardia* in-house database at Chiba University Hospital
