# Supplementary material for: Identification of Nocardia species using matrix-assisted laser desorption/ionization–time-of-flight mass spectrometry
Source: Clin Proteomics. 2015 Mar 7;12(1):6. doi: 10.1186/s12014-015-9078-5 (PMC4409724; doi:10.1186/s12014-015-9078-5)
Supplement: Additional file 1: Figure S5. — A spectra-based dendrogram of the manufacturer’s database strains and the 192 MMRC Nocardia isolates. The BioTyper (ver. 3.1) software was used to perform a cluster analysis and draw the dendrogram. The manufacturer’s database strains are displayed in red, and the MMRC isolates are displayed in black. [file 12014_2015_9078_MOESM1_ESM.ppt]

## Slide 1
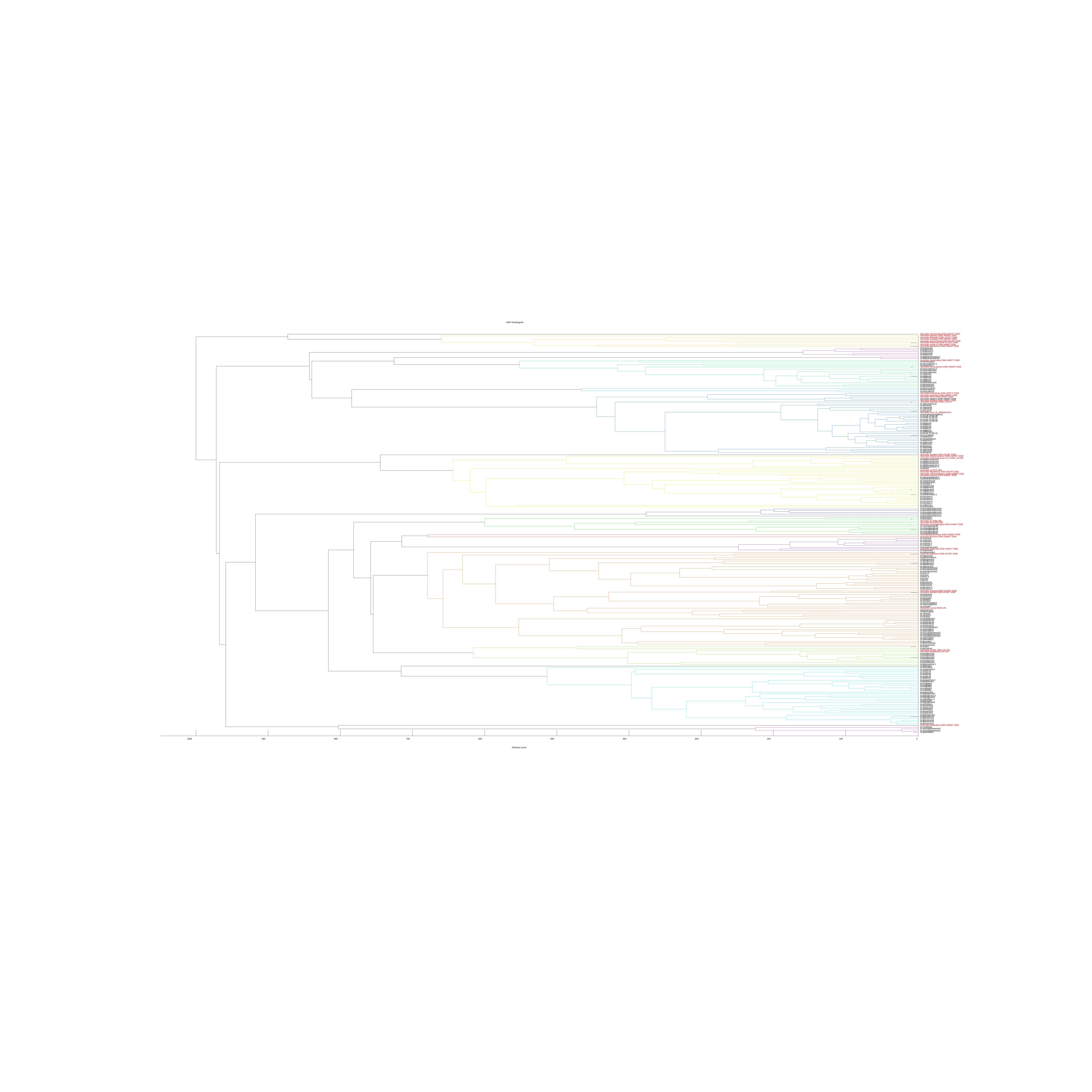

MSP Dendrogram
Nocardia salmonicida DSM 40472T DSM
Nocardia ignorata DSM 44496T DSM
Nocardia arthritidis DSM 44731T DSM
Nocardia exalbida DSM 44883T DSM
Nocardia pneumoniae DSM 44730T DSM
Nocardia araoensis DSM 44729T DSM
Nocardia asiatica DSM 44668T DSM
Nocardia abscessus DSM 44432T DSM
N.terpenica4
N.terpenica3
N.terpenica-2
N.terpenica5
N.terpenica1
N.diaphanozonaria-1
N.diaphanozonaria1
Nocardia vermiculata DSM 44807T DSM
N.vermiculate1
N.transvalensis-1
N.mexicana-1
Nocardia transvalensis DSM 43405T DSM
N.transvalensis-2
N.transvalensis5
N.transvalensis4
N.wallacei4
N.wallacei2
N.wallacei5
N.wallacei3
N.wallacei1
N.transvalensis3
N.blacklokiae2
N.blacklokiae1
N.paucivorans1
N.brericatena2
N.brericatena1
Nocardia kruczakiae DSM 44877T DSM
Nocardia aobensis DSM 44805T DSM
Nocardia nova DSM 44481T DSM
Nocardia elegans DSM 44890T DSM
Nocardia africana DSM 44491T DSM
Nocardia veterana RVA1_03 SLT
N.violaceofusca1
N.africana4
N.veterana2
N.veterana1
N.africana3
Nocardia nova 10_368938 MVO
N.pseudosporangifera1
N.nova5.13.06.19
N.nova4.13.06.19
N.nova3.13.06.19
N.nova1.13.06.19
N.elegans4
N.elegans2
N.elegans5
N.elegans3
N.elegans1
N.aobensis5
N.nova2.13.06.19
N.kruczakiae1
N.aobensis4
N.cerradoensis1
N.aobensis3
N.aobensis2
N.aobensis1
N.africana2
N.veterana4
N.veterana5
N.veterana3
N.africana1
Nocardia seriolae DSM 44129T DSM
Nocardia otitidiscaviarum DSM 43242T DSM
Nocardia otitidiscaviarum VA_01844_09 ERL
N.otitidiscaviarum5
N.otitidiscaviarum4
N.otitidiscaviarum3
N.otitidiscaviarum-2
N.otitidiscaviarum-1
N.italica1
Nocardia sp N133 IBS
Nocardia niigatensis DSM 44670T DSM
Nocardia yamanashiensis DSM 44669T DSM
Nocardia concava DSM 44804T DSM
N.yamanashiensis1
N.yamanashiensis-2
N.inohanensis2
N.inohanensis1
N.seriolae-1
N.niigatensis5
N.niigatensis2
N.niigatensis4
N.niigatensis3
N.niigatensis1
N.tsunamiensis-1
N.concava-3
N.concava-2
N.concava-5
N.concava-4
N.concava-1
N.uniformis1
N.acidivorans
N.pseudobrasiliensis4
N.pseudobrasiliensis5
N.pseudobrasiliensis3
N.pseudobrasiliensis2
N.pseudobrasiliensis1
N.fluminea2
N.fluminea-1
Nocardia sp N394 IBS
Nocardia sp N1064 IBS
Nocardia cyriacigeorgica DSM 44484T DSM
N.cyriacigeorgica5
N.cyriacigeorgica2
N.cyriacigeorgica3
N.cyriacigeorgica4
N.cyriacigeorgica1
Nocardia paucivorans DSM 44386T DSM
Nocardia farcinica DSM 43665T DSM
N.vinacea4
N.vinacea3
N.vinacea-5
N.vinacea-2
N.vinacea-1
N.pseudovaccinii1
Nocardia anaemiae DSM 44821T DSM
N.anaemiae1
N.salmonicide1
Nocardia higoensis DSM 44732T DSM
N.higoensis1
N.polyresistens1
N.lijiangensis1
N.takedensis3
N.takedensis2
N.takedensis1
N.speluncae1
N.shimofusensis3
N.shimofusensis2
N.shimofusensis1
N.puris-5
N.puris4
N.puris-3
N.puris2
N.puris1
N.farcinica4
N.farcinica-5
N.farcinica3
N.farcinica-2
N.farcinica-1
Nocardia testacea DSM 44765T DSM
Nocardia sienata DSM 44766T DSM
N.testacea3
N.testacea2
N.testacea1
N.sienate2
N.sienate1
N.rhamnosphilis2
N.rhamnosphilis1
N.carnea4
Nocardia carnea B334 UFL
N.jinanensis1
N.flavorosea1
N.carnea2
N.carnea-2
N.carnea1
N.caishijiensis1
N.thailandica4
N.thailandica3
N.thailandica2
N.thailandica1
N.novocastrense1
N.asteroides4
N.asteroides2
N.neocaledoniensis3
N.neocaledoniensis1
N.neocaledoniensis4
N.asteroides5
N.asteroides1
N.ignorata1
N.pneumoniae1
N.amamiensis1
N.ninae1
N.devorans1
Nocardia sp MB_9090_05 THL
Nocardia brasiliensis 251 RLT
N.brasiliensis2
N.brasiliensis5
N.brasiliensis4
N.brasiliensis3
N.brasiliensis1
N.tenerifensis1
N.altamirensis-1
N.harenae1
N.arthritidis5
N.xishanensis1
N.asiatica3
N.asiatica2
N.asiatica5
N.asiatica4
N.asiatica1
N.jiangxiensis-1
N.gamkensis1
N.exalbida4
N.exalbida5
N.exalbida3
N.exalbida2
N.exalbida1
N.araoensis2
N.beijingensis5
N.beijingensis-3
N.beijingensis2
N.artthritidis--2
N.arthritidis4
N.beijingensis4
N.arthritidis1
N.araoensis5
N.araoensis4
N.arthritidis3
N.araoensis3
N.araoensis1
N.beijingensis1
N.abscessus3
N.abscessus2
N.abscessus5
N.abscessus4
N.abscessus1
Nocardia thailandica DSM 44808T DSM
N.coubleae1
N.neocaledoniensis5
N.neocaledoniensis2
N.asteroides3
1000
900
800
700
600
500
400
300
200
100
0
Distance Level
