# Supplementary material for: Identification of Nocardia species using matrix-assisted laser desorption/ionization–time-of-flight mass spectrometry
Source: Clin Proteomics. 2015 Mar 7;12(1):6. doi: 10.1186/s12014-015-9078-5 (PMC4409724; doi:10.1186/s12014-015-9078-5)
Supplement: Additional file 2: Table S1. — A full detail of identity and characteristics of the 256 Nocardia isolates used in the study. Table S2. Improvement in the identification score by incorporating silica beads. Table S3. Comparison of identification scores from specimens extracted using the ethanol-formic acid extraction method (EFAE) and high-temperature extraction (HTEM) method. [file 12014_2015_9078_MOESM2_ESM.doc]

**Supplementary Figure Legends**

**Supplementary Figure 1. Comparison of representative mass spectra obtained from extraction with and without silica beads (*N. otitidiscaviarum* isolate).**Identification scores determined from each spectrum are indicated.

**Supplementary Figure 2. Comparison of representative mass spectra and identification scores for the same isolate extracted using the HTEM and EFAE methods (*N. farcinica* isolate).**
Specimens extracted using either the ethanol-formic acid extraction method (EFAE) or the high-temperature extraction method (HTEM) are compared.

**Supplementary Figure 3. Comparison of representative mass spectra and identification scores for the same isolate extracted using the HTEM and EFAE methods (*N. cyriacigeorgica* isolate).**Specimens extracted using either the ethanol-formic acid extraction method (EFAE) or the high-temperature extraction method (HTEM) are compared. A baseline rise due to noise was observed between 2,000 to 6000 m/z when the isolate was extracted using the HTEM method, and circled by red line (A). On the other hand, such rise was not observed when using the EFAE method (B). Each mass spectra matching between the spectrum collected and a reference spectrum stored in the database is indicated at the right top corner of each raw spectrum. Blue indicates the spectrum stored in the database used for pattern matching; in the upper half of the spectrum, green indicates matched peaks, red mismatched peaks, and yellow intermediate peaks. Like the baseline noise, mismatched peaks were observed between 2,000 to 6000 m/z when the isolate was extracted using the HTEM method, which may result in a decrease of identification score.

**Supplementary Figure 4. Comparison of representative mass spectra and identification scores for different extraction durations using the EFAE method.**Extraction durations of 1, 5, and 10 minutes in the EFAE (ethanol-formic acid extraction) method were tested with the same *N. nova* isolate.

**Supplementary Figure 5. A spectra-based dendrogram of the manufacturer’s database strains and the 192 MMRC *Nocardia* isolates.**

The BioTyper (ver. 3.1) software was used to perform a cluster analysis and draw the dendrogram. The manufacturer’s database strains are displayed in red, and the MMRC isolates are displayed in black.

**Supplementary Figure 6. Representative improvement in mass spectra matching and identification scores using the newly developed extraction method (NECLC) and the in-house database (NDCUH) to identify the same isolates (*N. nova*, *N. elegans*).**

With the introduction of the NECLC method and NDCUH database, the identification score for *N. nova* increased (A), and *N. elegans*, a strain that was previously unidentifiable, could now be identified to the species level (B). This figure shows matching between the spectrum collected and a reference spectrum stored in the database. Blue indicates the spectrum stored in the database used for pattern matching; in the upper half of the spectrum, green indicates matched peaks, red mismatched peaks, and yellow intermediate peaks.

Supplementary Tables

Supplementary Table 1 **A full detail of identity and characteristics of the 256 *Nocardia* isolates used in the study.**

|  | From the MMRC | | From the DCLC |
| --- | --- | --- | --- |
|  | Used in constructing the | Used in validation | |
|  | in-house database |  |  |
|  | No. of isolates | | |
| *Nocardia abscessus* | 5 | 1 | 1 |
| *Nocardia acidivorans* | 1 |  |  |
| *Nocardia africana* | 4 |  |  |
| *Nocardia altamirensis* | 1 |  |  |
| *Nocardia amamiensis* | 1 |  |  |
| *Nocardia anaemiae* | 1 |  |  |
| *Nocardia aobensis* | 5 | 2 |  |
| *Nocardia araoensis* | 5 |  |  |
| *Nocardia arthritidis* | 5 | 2 |  |
| *Nocardia asiatica* | 5 | 2 | 1 |
| *Nocardia asteroides* | 5 | 2 |  |
| *Nocardia beijingensis* | 5 | 2 |  |
| *Nocardia blacklokiae* | 2 |  |  |
| *Nocardia brasiliensis* | 5 | 2 |  |
| *Nocardia brevicatena* | 2 |  |  |
| *Nocardia caishijiensis* | 1 |  |  |
| *Nocardia carnea* | 4 |  |  |
| *Nocardia cerradoensis* | 1 |  |  |
| *Nocardia concava* | 5 | 2 |  |
| *Nocardia coubleae* | 1 |  |  |
| *Nocardia cyriacigeorgica* | 5 | 2 | 2 |
| *Nocardia devorans* | 1 |  |  |
| *Nocardia diaphanozonaria* | 1 |  |  |
| *Nocardia elegans* | 5 | 2 | 2 |
| *Nocardia exalbida* | 5 | 2 |  |
| *Nocardia farcinica* | 5 | 2 | 5 |
| *Nocardia flavorosea* | 1 |  |  |
| *Nocardia fluminea* | 2 |  |  |
| *Nocardia gamkensis* | 1 |  |  |
| *Nocardia harenae* | 1 |  |  |
| *Nocardia higoensis* | 1 |  |  |
| *Nocardia ignorata* | 1 |  |  |
| *Nocardia inohanensis* | 2 |  |  |
| *Nocardia italica* | 1 |  |  |
| *Nocardia jiangxiensis* | 1 |  |  |
| *Nocardia jinanensis* | 1 |  |  |
| *Nocardia kruczakiae* | 1 |  |  |
| *Nocardia lijiangensis* | 1 |  |  |
| *Nocardia Mexicana* | 1 |  |  |
| *Nocardia neocaledoniensis* | 5 |  |  |
| *Nocardia niigatensis* | 5 | 2 |  |
| *Nocardia ninae* | 1 |  |  |
| *Nocardia nova* | 5 | 4 | 4 |
| *Nocardia novocastrense* | 1 |  |  |
| *Nocardia otitidiscaviarum* | 5 | 1 | 2 |
| *Nocardia paucivorans* | 1 | 1 |  |
| *Nocardia pneumoniae* | 1 |  |  |
| *Nocardia polyresistens* | 1 |  |  |
| *Nocardia pseudobrasiliensis* | 5 | 2 |  |
| *Nocardia pseudosporangifera* | 1 |  |  |
| *Nocardia pseudovaccinii* | 1 |  |  |
| *Nocardia puris* | 5 | 2 | 1 |
| *Nocardia rhamnosphilis* | 2 |  |  |
| *Nocardia salmonicida* | 1 |  |  |
| *Nocardia seriolae* | 1 |  |  |
| *Nocardia shimofusensis* | 3 |  |  |
| *Nocardia sienata* | 2 |  |  |
| *Nocardia speluncae* | 1 |  |  |
| *Nocardia takedensis* | 3 |  |  |
| *Nocardia tenerifensis* | 1 |  |  |
| *Nocardia terpenica* | 5 |  |  |
| *Nocardia testacea* | 3 |  |  |
| *Nocardia thailandica* | 4 |  |  |
| *Nocardia transvalensis* | 5 | 2 |  |
| *Nocardia tsunamiensis* | 1 |  |  |
| *Nocardia uniformis* | 1 |  |  |
| *Nocardia vermiculata* | 1 |  |  |
| *Nocardia veterana* | 5 | 2 |  |
| *Nocardia vinacea* | 5 | 2 |  |
| *Nocardia violaceofusca* | 1 |  |  |
| *Nocardia wallacei* | 5 | 2 | 3 |
| *Nocardia xishanensis* | 1 |  |  |
| *Nocardia yamanashiensis* | 3 |  |  |
| Total | *192 | **43 | **21 |

*192 isolates from the MMRC were subjected to MALDI-TOF MS analysis to construct the in-house database, whereas 64 (**43 plus **21) isolates were used for validation of the in-house database.
MMRC = Medical Mycology Research Center, Chiba University.
DCLC = Department of Clinical Laboratory, Chiba University Hospital.

Supplementary Table 2. **Improvement in the identification score by incorporating silica beads.**

| Isolate | Without silica beads | With silica beads |
| --- | --- | --- |
| *N.otitidiscaviarum* | 1.889 | 2.100 |
| *N. cyriacigeorgica* | - | 1.250 |

The effect of the use of silica beads on the identification score was evaluated using a *N. otitidiscaviarum* isolate and a *N. cyriacigeorgica* isolate. Extracts of each of these isolates were independently spotted on 2 or more spots on the same target plate, subjected to MALDI analysis, and the scores were averaged.

Supplementary Table 3. **Comparison of identification scores from specimens extracted using the ethanol-formic acid extraction method (EFAE) and high-temperature extraction (HTEM) method.**

| Isolate | **EFAE method** | **HTEM method** |
| --- | --- | --- |
| *N. farcinica* | 1.462 | 1.444 |
| *N. cyriacigeorgica* | 1.715 | 1.600 |

The effect of the extraction method on the identification score was evaluated using a *N. farcinica* isolate and a *N. cyriacigeorgica* isolate. Extracts of each of these isolates were independently spotted on 2 or more spots on the same target plate, subjected to MALDI analysis, and the scores were averaged.

Supplementary Table 4. **Comparison of the identification scores from specimens extracted for different durations using the EFAE method.**

| Isolate | **1 min** | **5 min** | **10 min** |
| --- | --- | --- | --- |
| *N. nova* | 1.908 | 2.155 | 2.174 |
| *N. elegans* | - | - | 1.250 |

The effect of the duration of formic acid extraction on the identification score was evaluated using a *N. nova* isolate and a *N. elegans* isolate. Extracts of each of these isolates were independently spotted on 2 or more spots on the same target plate, subjected to MALDI analysis, and the scores were averaged. -: indicates sufficient mass spectra for scoring could not be obtained.
