# Supplementary figures and images for: Identification of Nocardia species using matrix-assisted laser desorption/ionization–time-of-flight mass spectrometry
Source: Clin Proteomics. 2015 Mar 7;12(1):6. doi: 10.1186/s12014-015-9078-5 (PMC4409724; doi:10.1186/s12014-015-9078-5)

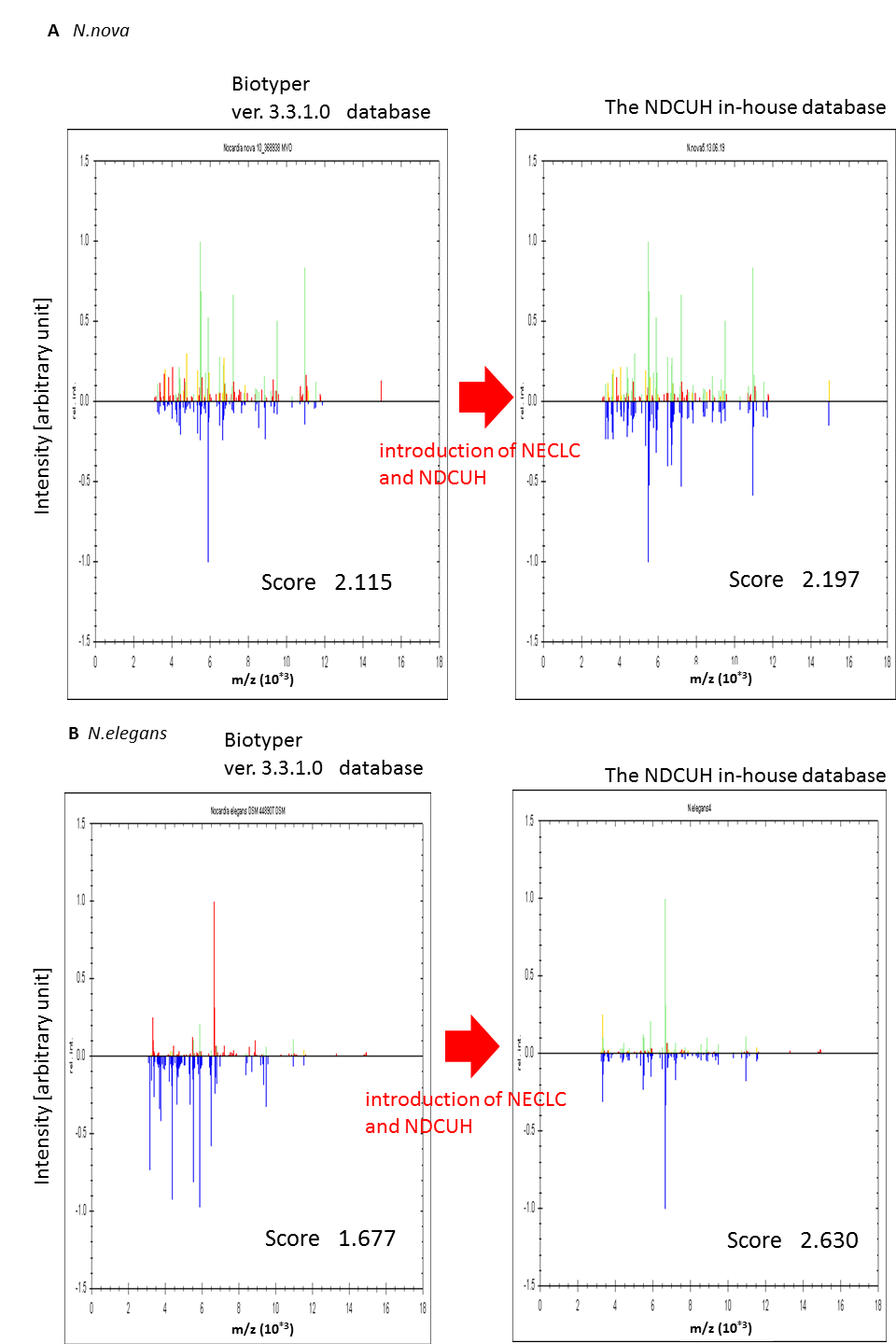

Supplement: Additional file 3: Figure S6. — Representative improvement in mass spectra matching and identification scores using the newly developed extraction method (NECLC) and the in-house database (NDCUH) to identify the same isolates (N. nova, N. elegans). With the introduction of the NECLC method and NDCUH database, the identification score for N. nova increased (A), and N. elegans, a strain that was previously unidentifiable, could now be identified to the species level (B). This figure shows matching between the spectrum collected and a reference spectrum stored in the database. Blue indicates the spectrum stored in the database used for pattern matching; in the upper half of the spectrum, green indicates matched peaks, red mismatched peaks, and yellow intermediate peaks. [file 12014_2015_9078_MOESM3_ESM.tiff]

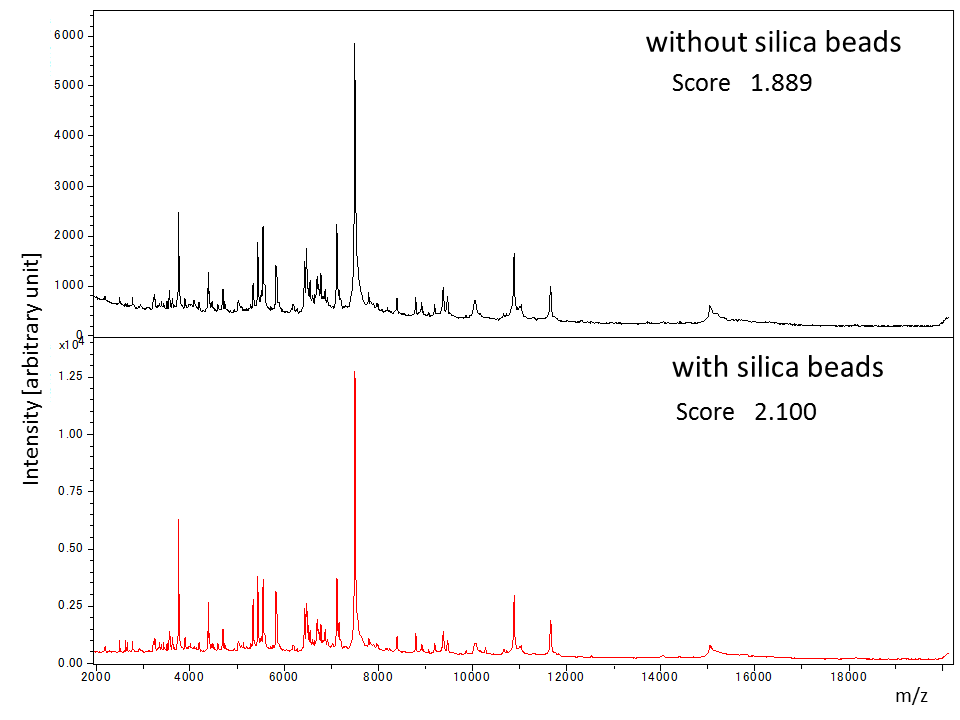

Supplement: Additional file 4: Figure S1. — Comparison of representative mass spectra obtained from extraction with and without silica beads (N. otitidiscaviarum isolate). Identification scores determined from each spectrum are indicated. [file 12014_2015_9078_MOESM4_ESM.tiff]

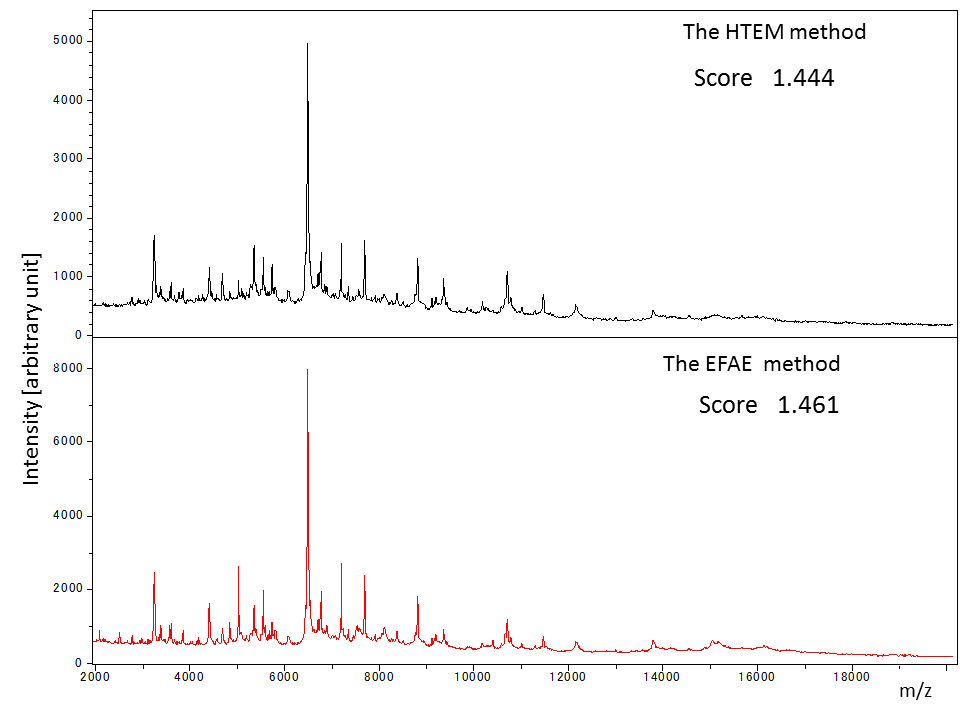

Supplement: Additional file 5: Figure S2. — Comparison of representative mass spectra and identification scores for the same isolate extracted using the HTEM and EFAE methods (N. farcinica isolate). Specimens extracted using either the ethanol-formic acid extraction method (EFAE) or the high-temperature extraction method (HTEM) are compared. [file 12014_2015_9078_MOESM5_ESM.tiff]

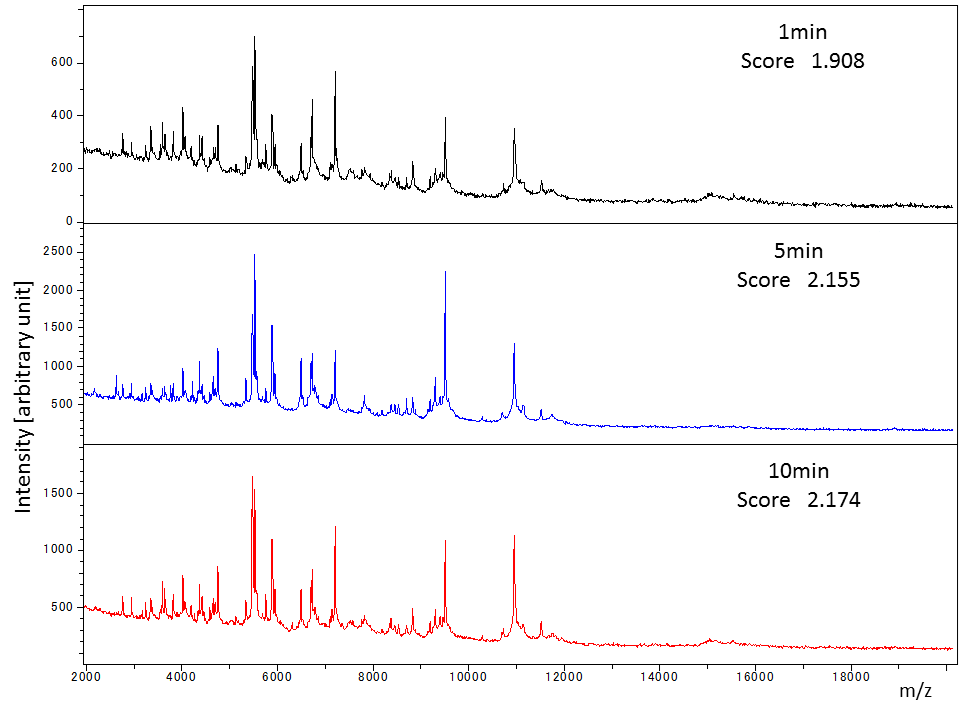

Supplement: Additional file 7: Figure S4. — Comparison of representative mass spectra and identification scores for different extraction durations using the EFAE method. Extraction durations of 1, 5, and 10 minutes in the EFAE (ethanol-formic acid extraction) method were tested with the same N. nova isolate. [file 12014_2015_9078_MOESM7_ESM.tiff]
