# Supplementary material for: Identification of Nocardia species using matrix-assisted laser desorption/ionization–time-of-flight mass spectrometry
Source: Clin Proteomics. 2015 Mar 7;12(1):6. doi: 10.1186/s12014-015-9078-5 (PMC4409724; doi:10.1186/s12014-015-9078-5)
Supplement: Additional file 6: Figure S3. — Comparison of representative mass spectra and identification scores for the same isolate extracted using the HTEM and EFAE methods (N. cyriacigeorgica isolate). Specimens extracted using either the ethanol-formic acid extraction method (EFAE) or the high-temperature extraction method (HTEM) are compared. A baseline rise due to noise was observed between 2,000 to 6000 m/z when the isolate was extracted using the HTEM method, and circled by red line (A). On the other hand, such rise was not observed when using the EFAE method (B). Each mass spectra matching between the spectrum collected and a reference spectrum stored in the database is indicated at the right top corner of each raw spectrum. Blue indicates the spectrum stored in the database used for pattern matching; in the upper half of the spectrum, green indicates matched peaks, red mismatched peaks, and yellow intermediate peaks. Like the baseline noise, mismatched peaks were observed between 2,000 to 6000 m/z when the isolate was extracted using the HTEM method, which may result in a decrease of identification score. [file 12014_2015_9078_MOESM6_ESM.ppt]

## Slide 1
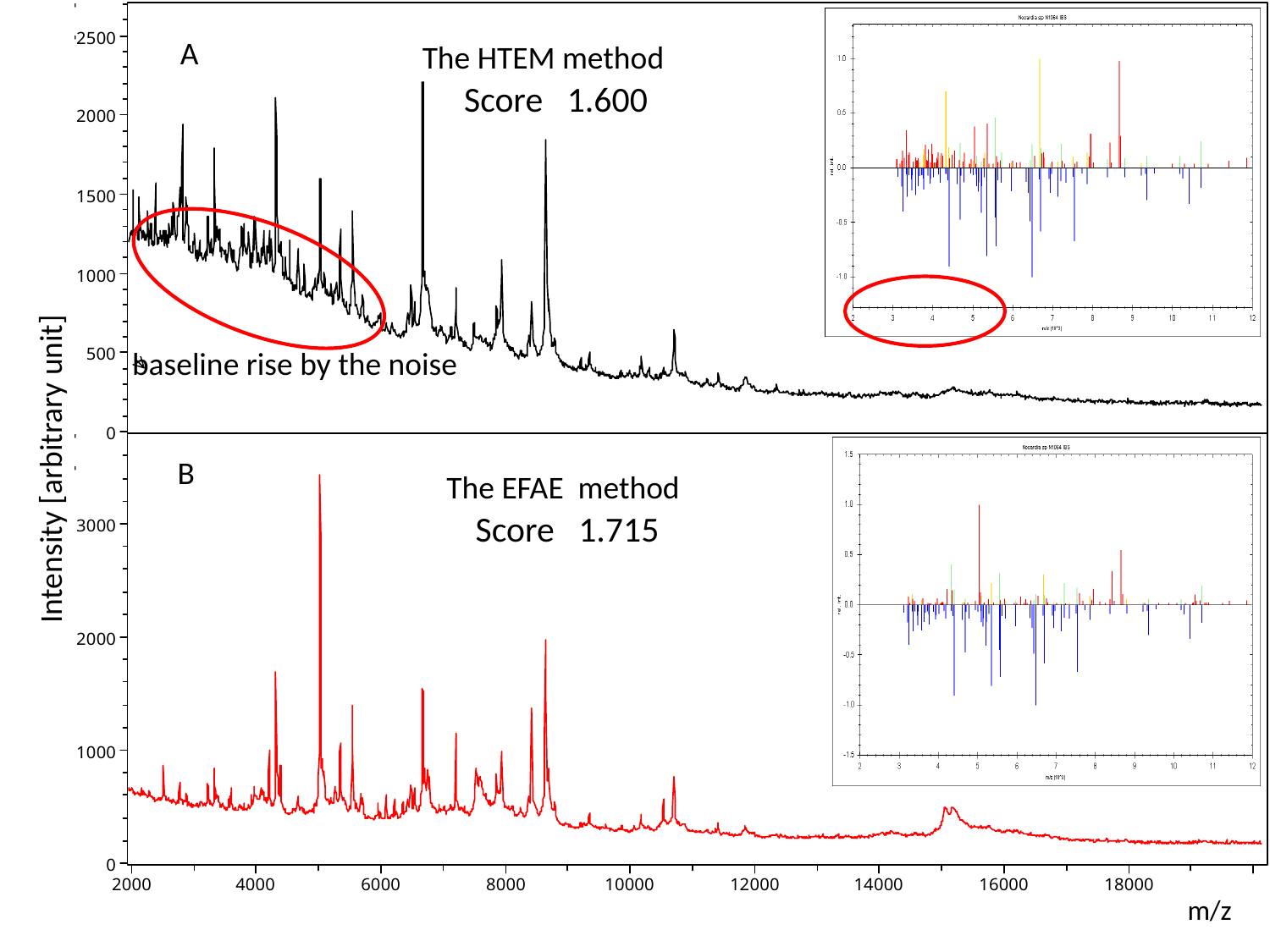

A
The HTEM method
Score 1.600
Intensity [arbitrary unit]
baseline rise by the noise
B
The EFAE method
Score 1.715
m/z
